# Mapping virtual platforms to estimate the population size of men who have sex with men (MSM) who use internet to find sexual partners: implications to enhance HIV prevention among MSM in Kenya

**DOI:** 10.12688/gatesopenres.13158.2

**Published:** 2020-12-10

**Authors:** Faran Emmanuel, Japheth Kioko, Helgar Musyoki, Shem Kaosa, Martin Kyana Ongaro, Samuel Kuria, Kennedy Olango, Janet Musimbi, Jeffrey Walimbwa, James Blanchard, Shajy Isac, Parinita Bhattacharjee

**Affiliations:** 1 Institute of Global Public Health, University of Manitoba, Winnipeg, Canada; 2 Partners for Health and Development in Africa, Nairobi, Kenya; 3 National AIDS and STI Control Programme, Ministry of Health, Nairobi, Kenya; 4 HIV and AIDS People’s Alliance of Kenya, Mombasa, Kenya; 5 Mamboleo Peer Empowerment Group, Kiambu, Kenya; 6 Men Against AIDS Youth Group, Kisumu, Kenya; 7 G10 Research Network, Nairobi, Kenya; 8 India Health Action Trust, Delhi, India

**Keywords:** MSM, Virtual platforms, GSN platforms, Size estimation, Kenya

## Abstract

**Introduction: **Men who have sex with men (MSM) in Kenya face a disproportionate HIV disease burden. Over the last few years, the use of virtual platforms and internet sites to seek male sexual partners has increased manyfold in Kenya. New approaches are required to map, estimate and profile MSM who operate through virtual platforms to design interventions for them.

**Methods:** This study was conducted in three counties in Kenya: Kiambu, Kisumu and Mombasa with MSM who use virtual platforms such as geosocial networking (GSN) and social networking applications to find and connect with male sex partners. The platforms were profiled through a multi-stage approach and the number of MSM associated with these platforms were estimated. In the final stage, 435 respondents randomly selected from the virtual platforms were interviewed in a secure location after informed consent. Data analysis focused on calculating an estimate of MSM for each virtual platform in each site, adjusting for duplicate profiles and multiple registrations.

**Results:** We identified 24 GSN apps, 18 Facebook accounts/pages and 18 WhatsApp groups across the three counties, with Facebook being the preferred platform. Kiambu had the highest number of estimated MSM at 3,635 (95%CI = 3,335 to 3,990) followed by Kisumu at 1,567 (95%CI = 1,480 to 1,665) and Mombasa at 1,469 (95%CI = 1,335 to 1,604) who used virtual platforms to find other male sexual partners. On average, each MSM had 3.7 profiles on multiple platforms, with an average of 2.1 profiles used in the past month.

**Conclusions:** The use of conventional population size estimation approaches that focus on physical sites alone may underestimate the total number of MSM in a geography. Virtual mapping should be used in conjunction with conventional hot spot based size estimation methodologies to estimate numbers of MSM to set programmatic targets.

## Introduction

Kenya has the joint third largest epidemic in the world, with over 1.6 million people living with HIV ^1^. Kenya is characterized as having a generalized epidemic among the adult population; however, key populations including female sex workers, men who have sex with men (MSM) and people who inject drugs are at a heightened risk of HIV acquisition and transmission due to their sexual and social behaviors ^2, 3^. MSM carry a disproportionate burden of HIV in Kenya, with a HIV prevalence of 18.2%, compared to a HIV prevalence of 3.1% among adult Kenyan men (including MSM), and contribute to nearly 15% of new infections each year ^3– 5^. In Kenya, “MSM” encompass a range of sexual identities including gay, bi-sexual, heterosexual and men who sell sex to other men ^6^. To scale up HIV prevention, there is a need to estimate the size of key populations and also understand where they congregate and can be reached ^7^. Programmatic mapping and size estimation of MSM conducted by NASCOP in 2018 estimated a total number of 32,580 MSM (ranging between 24,704 to 40,455) in 34 counties in Kenya ^8^. Since discriminatory legislation, criminalization and stigma attached to same-sex relationships poses major barriers for MSM to seek other male partners at physical sites, estimates obtained from conventional size estimation approaches including geographic and programmatic mapping are considered to underestimate of the total number of MSM ^9^.

Globally, with recent advances in information technology and improved mobile networks, an increased number of MSM have started using the internet and geosocial networking mobile phone applications (GSN apps) to seek and meet other male sexual partners ^10– 12^. A meta-analysis of internet use among MSM concluded that approximately 35% to 45% of MSM use social media to exchange information, socialize and support each other ^13^. A recent study conducted among MSM in Kenya found that 64% of the respondents used the internet and virtual platforms to seek male sexual partners ^14^. These include GSN apps such as Grindr, Scruff, Adam4Adam, Gay Exchange etc., as well as social media applications such as Facebook and general communication apps like WhatsApp. GSN apps use geo-location features of smart phones to display potential contacts based on their physical proximity, making sexual partner seeking quick and convenient ^15^. Through these apps, users create individual profiles with pictures and are able to view people online within their geographical proximity ^16^. Other social media apps such as Facebook and messenger apps such as WhatsApp are messaging and calling platforms available for free, provided internet is available ^17^. Through these networks one could connect with people they already know or could join a local/international network or group through an invitation or a link.

As same sex relationships are stigmatized, the internet provides a safe space for MSM to connect and find sexual partners in a covert manner without facing stigma, discrimination and negative reactions from the larger community ^18^. These virtual platforms act like a safe community space for MSM and that has led to a decline in the number and significance of public locations where MSM meet other men, especially in countries where same sex relationships are criminalized ^9^. Given the increasing popularity of the internet to find sexual partners, compounded by discriminatory laws and social stigma specifically affecting MSM populations, it is more challenging to enumerate their numbers and estimate population size and expand HIV prevention services to MSM engaged in these virtual sites ^19, 20^. While there has been research conducted to understand the MSM using virtual sites, the studies have mostly looked at risk behaviors and operations of this dynamic sub-typology ^12, 13, 15^. Indirect estimation of the population size of MSM has also been attempted; however, such estimation provides more generalized estimates based on the number of MSM registered on various sites ^11^. In this paper we present an emerging new approach called virtual mapping to locate and estimate the population size of MSM who seek male sexual partners through the internet and various phone-based applications. The approach has been tested and utilized with reliable results in a few countries and was modified to the local context in Kenya.

## Methods

### Study design

The data collection was done as part of the routine HIV prevention programming with MSM in the three counties, Kisumu, Mombasa and Kiambu, in Kenya. The three counties represent three distinct geographies in Kenya with an HIV prevalence between 9% to 27%. Kiambu is located in the central, Kisumu is located in the western part and Mombasa is in the coastal part of Kenya ^21^. In these counties, the prevention program is being implemented by MSM-led community-based organizations (CBOs). Mapping of virtual sites where MSM meet other sexual partners and cross-sectional data collection from respondents selected from the virtual sites was done by CBO members and program staff. Secondary analysis of this data was carried out to write this paper.

### Study setting

The community-led data collection was conducted in three counties, namely Kiambu, Kisumu and Mombasa, which were selected based on a high number of MSM as well as a high prevalence of HIV, at 23%, 13% and 19% in Kiambu, Kisumu and Mombasa, respectively ^21^. The study was led by the University of Manitoba and National AIDS and STI Control Program (NASCOP) in partnership with three MSM-led community-based organizations (CBOs), namely Mamboleo Peer Empowerment Group (MPEG) in Kiambu, Men Against AIDS Youth Group (MAAYGO) in Kisumu and the HIV & AIDS People’s Alliance of Kenya (HAPA Kenya) in Mombasa. The study received technical support from the G10 MSM research network in Kenya. Field data collection was conducted from June to July 2018 by 12 MSM community researchers known as virtual mappers (four in each site), who were trained in a three-day training prior to the study.

### Data collection

The study used a three-stage approach to map and estimate the population size of MSM.

Stage I served as the first step, with the aim of developing a comprehensive list of all virtual platforms and sites used by MSM to find sexual partners in the three geographies. A diverse group of virtual mappers were recruited. These virtual mappers were members of the three MSM-led CBO’s, some of them were students and others worked as peer educator and outreach workers in the CBO and were well versed in the use of the internet. They reached out and consulted other MSM community members in their social network and developed a comprehensive list of all WhatsApp groups, Facebook pages and GSN applications in use within the selected counties.

Stage 2 involved profiling each of these virtual platforms/sites identified in the previous stage, including validating its active functioning and estimating the size of MSMs using it at different timings. Virtual mappers created their own profiles and regularly visited each platform/site at three different times of the day for a period of two weeks based on a schedule developed by the investigators. They used a pre-designed format to note down the day and time of their visit to each platform and internet site, the total number of registered users within the county’s catchment area and the number of MSM online at that particular time. Information on the total number of registered MSM within the catchment area visible while online was used to estimate the population size of MSM.

In stage 3, interviews were conducted with randomly selected MSM from multiple virtual sites in each county. To achieve an optimum sample size, we assumed a population prevalence of 50% with a precision of 5% and 95% confidence interval. Since we expected a higher non-response, we inflated the sample size calculated by 15%, which calculated an overall sample of 440. To achieve this sample, we randomly selected 15 virtual sites in each county and a random sample of 10 MSM from each selected platform were selected using a random number table from all users who were online, on different days and at different times. The virtual mapper logged on, randomly selected a person, introduced the study and invited the selected person to participate in the face-to-face survey following online informed consent. If the person agreed to participate, he was invited at a convenient time and place for a face-to-face interview as mutually agreed. The interviews were conducted by the 12 virtual mappers in a secure room, in a secure office building, mostly at the drop-in centre of the MSM led organization, after written consent was obtained. These data collectors were trained and monitored regularly by the University of Manitoba’s team during the data collection process. Data quality checks were done on a regular basis and any data errors were quickly identified and rectified by the study coordinators/data managers. The interviewers used a standard close ended questionnaire ^22^ and each interview took 25–30 minutes. A total number of 435 MSM were interviewed (119 in Kiambu, 172 in Kisumu and 144 in Mombasa).

### Data management and analysis

Data were collected using a structured questionnaire ^22^, which was reviewed and revised by the virtual mappers before handing it over to the data management team. During the revisions the mappers reviewed the relevance of each question, language used and sensitivity of the MSM community towards the question. The information was entered into a database specifically designed for this study in Microsoft Excel for stage 1 and stage 2 data and SPSS 25.0 for stage 3 data. The analysis was conducted using SPSS 25.0. Stage 2 data provided the total number of users registered with each virtual platform and an unadjusted estimate of registered MSM within each county was calculated. This unadjusted estimate included duplicates owing to the use of multiple platforms as well as multiple registrations on a single platform. Questionnaire information gathered during face-to-face interviews in stage 3 provided correction factors that allowed us to adjust for this duplication using the number of profiles each MSM could have. We used a correction factor accounting for the mean number of profiles for each MSM, as well as the proportion that uses multiple platforms and sites. The correction factor was used to adjust for duplication and calculate the estimated number of MSMs in each county using the following formula;

$$E=\sum_{i=1}^{n}\text{Ei}*\left(\text{1-p}\right)+\sum_{i=1}^{n}\text{Ei}*\frac{\text{p}}{\text{m}}$$

Where *E* is the adjusted estimate of all MSM registered at the virtual site; *Ei* is the number of MSMs registered with virtual platform *i*; *p* is the proportion of MSMs using more than one virtual platform; *m* is the mean number of virtual platforms an MSM used; and *i=1…n* is the number of virtual platforms. Once an estimate was developed, we created a 95% confidence interval around the estimated number of MSM in each county as;

Lower estimate of MSM (E _L_), E _L_ = $\sum_{i=1}^{n}\text{Ei}*\left(\text{1-p}\right)+\sum_{i=1}^{n}\text{Ei}*\left(\frac{\text{p}}{m_{u}}\right)$ and the upper estimate of MSM (E _U_), E _U=_ $\sum_{i=1}^{n}\text{Ei}*\left(\text{1-p}\right)+\sum_{i=1}^{n}\text{Ei}*\left(\frac{\text{p}}{m_{l}}\right)$, where m _l_ and m _u_ are the lower and upper confidence limit of mean number of profiles.

### Ethical considerations

Ethical approval for secondary analysis of the study data was obtained from the Ethical Review Committee of Kenyatta National Hospital, University of Nairobi (P647/11/2017). International ethical guidance was followed to maintain confidentiality of participants i.e., no recording of participant identity or personal identification information, use of unique identifying codes, use of password protected electronic data files, and limiting access to the data files to authorized individuals only. Informed written consent was obtained, and all interviews were conducted in a safe and secure place. All participating MSM were compensated for their time and travel and were paid a compensation in Kenyan Shillings equivalent to $5 USD. Debriefing sessions were conducted after the interviews and all participating MSM were referred to HIV prevention, treatment and care facilities.

## Results

Table 1 presents information collected for various virtual platforms and internet sites used by MSM in the study counties. We identified a total number of 60 platforms classified within three broader types i.e., 24 GSN apps, 18 Facebook pages and 18 WhatsApp groups. Among the GSN sites, Badoo, Grindr, Tagged, Planet Romeo and Hornet were the five most used apps in all three counties. The majority (79%) of the MSM knew of a virtual platform other than the one they primarily used to find partners. Nearly one-fifth (19.3%) of MSM had only one registered profile, 62% had two to four user profiles on different virtual platforms and 15.4% had more than four user profiles. On average, each MSM had 3.7 registered profiles on multiple virtual platforms. Analyzed by type, those using WhatsApp had 5.6 profiles on multiple sites, followed by GSN app users and Facebook users who had 3.8 and 2.7 profiles, respectively. While a high number of profiles were reported, approximately two profiles were reported to be used by respondents in the last month. Nearly three-quarters (74%) of respondents reported that they also visited physical spots, with a higher percentage of those who visited being from WhatsApp groups (86%). However, 26% of the respondents reported exclusively using virtual platforms. Overall, we estimated a total number of 6,672 (95%CI = 6,174 to 7,259) MSM registered on all virtual platforms included in this study. Facebook had the highest number of registered MSM at 5,910 (95%CI = 5,076 to 7,072), followed by GSN sites (3,118; 95%CI = 2,937 to 3,324) and WhatsApp (746; 95%CI = 642 to 891).

**Table 1.** Virtual platforms used by MSM in selected counties in Kenya, 2018.

Variable	Total	Type of virtual platforms/site		
		GSN	Facebook	WhatsApp
Total number of platforms identified	60	24	18	18
Used another virtual platform in addition to the primary platform used	79.1 %	82.4 %	63.1 %	93.8 %
Total number of profiles ^*^				
* • Single profile*	19.3 %	16.9 %	32.0 %	6.3 %
* • 2 to 4 profiles*	61.8 %	62.0 %	51.5 %	83.3 %
* • More than 4 profiles*	15.4 %	18.3 %	9.7 %	10.4 %
Average number of registered profiles	3.7	3.8	2.7	5.6
Profiles used in the last month	2.0	2.1	2.0	1.7
MSM who also visited physical spots to find partners (%)	74 %	71 %	78 %	86 %
Number of MSM estimated on each virtual platform				
• Total estimated number of MSM (95% CI)	6,672 (6174- 7259)	3,118 (2937- 3324)	5,910 (5076- 7072)	746 (642- 891)
• Average ± SD number of MSM on each site	215 ± 199	209 ± 60	391 ± 587	46 ± 22

* No of accounts created by each respondent. MSM, men who have sex with men; GSN, geosocial networking.

Table 2 presents the estimated number of MSM who operate through virtual platforms by county. Results show that MSM in Mombasa used the most virtual platforms (43) followed by those in Kiambu (34) and Kisumu (29). Kiambu had the highest number of estimated MSM, calculated at 3,635 (95%CI = 3,335 to 3,990), followed by Kisumu at 1,567 (95%CI = 1,480 to 1,665) and Mombasa at 1,469 (95%CI = 1,335 to 1,604).

**Table 2.** Estimated number of MSM operating through virtual platforms in selected counties in Kenya, 2018.

	Total	Kiambu	Kisumu	Mombasa
Virtual platforms identified ^*^				
* • GSN apps*	24	22	22	23
* • Facebook*	18	7	1	10
* • WhatsApp*	18	5	6	10
Estimated number of MSM				
* • Average*	6,672	3,635	1,567	1,469
* • Min*	6,174	3,335	1,480	1,335
* • Max*	7,259	3,990	1,665	1,604

* County platforms don’t add to the overall total because same platforms exist across counties. MSM, men who have sex with men; GSN, geosocial networking.

Socio-demographic characteristics and sexual profiles of MSM using various virtual platforms disaggregated by county as well as by the type of virtual platform used are shown in Table 3. Of the 435 respondents interviewed, 61% were below the age of 25 years. Kisumu had a high proportion (68%) of respondents who were under 25 years of age compared to Kiambu (61%) and Mombasa (51%). No age specific differences were noted between MSM using various platforms or sites. The majority of respondents had completed at least secondary education. In terms of sexual orientation, 69% of respondents self-identified as gay men, while 30% identified as bisexual. A large proportion of the respondents, 83% in Mombasa and 79% in Kiambu, identified themselves as gay men compared to 52% in Kisumu. A higher proportion of respondents (77%) using Facebook identified themselves as gay, followed by GSN apps (68%) and WhatsApp (60%). The majority of respondents (97%) identified themselves as male, with 3% identifying themselves as transgender. Almost half (48%) of respondents in Kisumu reported assuming the top sexual role compared to 40% in Mombasa and 34% in Kiamabu. Similarly, 63% of MSM on WhatsApp reported to assume the top sexual role as compared to 39% on GSN sites and 38% on Facebook. The study also found that 69% of respondents sold sex for money as sex workers; Mombasa reported a large proportion of sex workers (70%) compared to Kisumu (60%) and Kiambu (45%). More respondents using WhatsApp (68%) reported selling sex for money compared to Facebook (65%) and GSN apps (56%).

**Table 3.** Socio-demographic, sexual & network related characteristics of MSM operating through virtual platforms in selected counties in Kenya, 2018.

Total MSM interviewed (N)	Total		County		Type of virtual platform		
		Kiambu	Kisumu	Mombasa	GSN app	Facebook	WhatsApp
	435	119	172	144	284	103	48
Age							
• *<25 years*	61%	61%	68%	51%	60%	63%	63%
• *25+ years*	39%	39%	32%	49%	40%	37%	38%
Education							
• *No formal education*	1%	None	3%	None	1%	2%	2%
• *Secondary*	53%	27%	48%	80%	47%	65%	60%
• *Tertiary/college*	46%	73%	48%	19%	51%	33%	38%
Sexual orientation							
• *Gay*	69%	79%	52%	83%	68%	77%	60%
• *Bisexual*	30%	21%	47%	17%	31%	23%	38%
Gender identity							
• *Male*	97%	98%	99%	94%	98%	96%	98%
• *Trans*	3%	2%	1%	6%	2%	4%	2%
Sexual role							
• *Bottom*	29%	33%	27%	29%	30%	30%	21%
• *Top*	42%	34%	48%	40%	39%	38%	63%
• *Versatile*	25%	32%	15%	31%	24%	31%	17%
Sells sex	59%	45%	60%	70%	56%	65%	68%
People connected in last week	16.8	22.7	19.8	7.9	19.0	10.1	17.4
Men with whom had anal sex in last week	4.9	2.7	8.1	2.5	4.9	4.8	5.0
Men to whom sold sex last week	2.8	1.3	4.4	1.9	2.7	3.5	1.7
Men to whom sold sex last week (among those sold sex)	4.1	2.8	6.3	2.5	4.4	4.8	1.9

MSM, men who have sex with men; GSN, geosocial networking.

Respondents reported virtually connecting with 16.8 male partners in the last week, the highest being in Kiambu (22.7) compared to Kisumu (19.8) and Mombasa (7.9) and on GSN apps (19) compared to WhatsApp (17.4) and Facebook (10.1). The connections mentioned are only “digital connections” i.e, other MSM connected through virtual sites and apps and does not include MSM connected through physical sites”. Respondents reported an average of 4.9 men with whom they had anal sex in the last week and sold sex to an average of 2.8 men. County-wide analysis showed respondents in Kisumu had a significantly higher number of partners (8.1 and 4.4 with whom they had anal sex and sold sex, respectively) compared to Kiambu (2.7 and 1.3) and Mombasa (2.5 and 1.9) while no differences were noted in site-based analysis of sexual encounters in the last week. Similar differences were seen in the platforms/sites, with respondents using WhatsApp reporting having anal sex with five men in the last week and selling sex to 1.7 men compared to GSN platforms (4.9 and 2.7) and Facebook (4.8 and 3.5). When considered those selling sex only, on an average they sold sex to 4.1 persons as against 2.8 among the total respondents. Among the MSM selling sex, the number with whom they sold sex vary from 2.5 in Mombasa to 6.3 in Kisumu and 1.9 among WhatsApp group members to 4.8 among Facebook page users.

## Discussion

Although a virtual mapping approach has been used in a few countries ^23– 25^ it was used for the first time in Kenya and has successfully identified all key virtual platforms and internet sites, along with the estimated number of MSM who use these platform/sites to find male sexual partners. Based on how each platform or site operates, we classified them into three broader types. The first is GSN apps (e.g., Badoo, Grindr, Hornet), which require GPS-enabled smart phones, and allow subscribers to register profiles with personal information, upload pictures, share their location and see other network members within a specific distance. The second variant is social media applications like Facebook and, finally, communication applications such as WhatsApp. The latter two are general purpose social networking platforms, have a larger generalized use and are not uniquely designed for seeking partners. Interestingly, we found several designated Facebook pages and WhatsApp groups specifically created for the purpose of finding MSM sexual partners even though these sites needed invitation or acquaintance with an existing member of these groups to join. These WhatsApp and Facebook groups were local to the counties in which this study was conducted. WhatsApp and Facebook groups have no geo-spatial info for the participants, which is a feature only of GSN apps. Our finding that Facebook was the most used virtual platform by MSM to seek male sexual partners is also in agreement with previous research ^26^. The MSM estimates developed through this study may be much more realistic and closer to the actual number of MSM population in Kenya. The previous MSM estimates were derived using geo-spot based programmatic mapping techniques and estimated 1,664 MSM in Kiambu, 2,492 in Kisumu and 2,855 in Mombasa ^8^. Since that methodology did not include MSM who use virtual platforms, there could have been an underestimation of the total number of MSM in Kenya. Although our study focused on MSM who use virtual platforms and the internet and estimated 3,635 MSM in Kiambu, 1,567 MSM in Kisumu and 1,469 MSM in Mombasa, we found a significant overlap between MSM who use virtual sites and those who go to physical spots to find new partners. Nearly three-quarters of the MSM mainly using virtual platforms also visited physical spots to find partners, which is in concurrence with another survey conducted among MSM in these three counties. The survey showed that 14.7% of the MSM sought male sexual partners only in physical sites, 64.0% in both physical and virtual sites and 21.2% in only virtual sites ^14^. These findings warrant the importance of conducting mapping of both physical and virtual platforms, as developing size estimates from a single approach may underestimate the number of MSM. At the same time, this presents another important insight that the overall estimate of MSM isn’t a simple additive function of geo-spot based MSM and virtual mapping estimates, but would require an adjustment of the overlapping proportion of MSM who use both physical and virtual sites. When adjusted for these overlaps, the total number of MSM in Kenya were approximately 25% in excess of the estimated number of MSM derived through geo-mapping.

We also calculated the standard measure of per capita i.e., number of online MSM per 1000 adult (15–64 years) males. It shows that the per capita MSM online per 1000 adult males are 6.7, 7.2 and 4.1 for Kiambu, Kisumu and Mombasa respectively. In addition to providing population size estimates, the current study has also enhanced our understanding of virtual platform and internet based MSM in terms of their profiles, networks and how they connect with each other. Our results show that the participants in this study are young males with high education levels, which has also been shown in previous studies ^10, 12^. This may be reflective of the access and ease of use of the internet within a specific segment of the population in a resource constrained country. We have also seen that more than 80% of MSM use multiple sites, and also have multiple identities registered on a single site. This is also in concurrence with previous research ^25– 27^. Estimating the size of this population based on total counts of registered MSM at various virtual sites, without adjusting for these duplications will lead to an over-representation of the population size manyfold more than the actual numbers ^11^.

The findings of this study have several implications for HIV programming for MSM in Kenya. A substantial proportion of MSM stay hidden and are unlikely to receive services regularly through the existing MSM programs. Missing this population from a HIV program would mean denying critical HIV prevention and treatment services to a very high risk and vulnerable sub-population of MSM. In Kenya, we previously found that MSM who operate through virtual sites alone had a much higher HIV prevalence (26.7%) compared to those who seek sexual partners in physical and virtual sites (15.4%) or only physical sites (8.5%) ^14^. Identifying MSM who engage in these virtual sites provides an avenue to reach them with HIV prevention and care services. HIV prevention programs should include virtual mapping in their strategic design and engage outreach workers and peer educators to reach out to these MSM. Those who wish to stay invisible and do not feel comfortable coming to the MSM led clinic or services can be offered outreach services at a safe space of their choice.

The findings of this study should be considered in light of a few limitations. Firstly, the accuracy of results is dependent on the accuracy of the app itself i.e., geo-specificity allowed by the GSN apps. Thus, the design of the study will not work appropriately in contexts where geo-specification of the catchment area is not allowed by the GSN apps. Sampling of respondents was also subject to a level of selection bias. Although the sample was based on a random selection of multiple sites, the selection of MSM was based on who was available at that moment and also who was willing to participate. The estimation of the population size of MSM is also based on the total number of MSM registered on various GSN apps and internet sites, and some registrations could have been redundant as well. Owing to a smaller sample size, the estimate generated have wider ranges, which could have been more precise if a larger sample size was achieved. Finally, our approach to virtual-site sampling made it challenging to document a non-response rate, which further limits our ability to judge selection bias. Although the methodology has limitations, it still provides a simple approach to estimate the number of MSM connected to virtual platforms in addition to understanding the operational dynamics of this concealed sub-typology which can be utilized to improve their reach and coverage.

## Conclusions

To conclude, as internet usage around the world increases, its use by MSM will continue to gain popularity to find sexual partners, especially in cultures where same sex relationships are stigmatized. There is an increasing need to understand this subgroup, its size and dynamics to plan, develop and implement evidence-based prevention programs. The research methodology presented in this paper was able to map various virtual platforms and internet sites used by MSM and provide a methodology to estimate their size. The approach is simple and pragmatic and could be utilized to immediately initiate interventions among MSM who operate through virtual networks and stay hidden from programs. Although the approach might have limitations, there is a clear indication that use of geo-spot based mapping alone underestimates the total number of MSM in a given context and therefore should be used in conjunction with this methodology to calculate population estimates, set programmatic targets and initiate interventions to reach hidden and hard to reach MSM.

## Data availability

### Underlying data

This data is confidential considering the fact that MSM are a criminalized population in Kenya and sharing names of sites may put their life in danger. Aggregate level de-identified data tables are available at http://www.phdaf.org/publications/ and on Harvard Dataverse (see below). The corresponding author ( bhattacharjee.parinita@gmail.com) will be able to facilitate access to the full underlying data. A formal request needs to be made and a data sharing agreement will have to be made before sharing the data.

Harvard Dataverse: Data for Virtual Mapping in Kenya. https://doi.org/10.7910/DVN/9B9FFB ^28^

This project contains the following underlying data:

- DATA MSM interviews.tab (aggregate data collected through Form C)
- DATA Virtual Site Visit profiling.tab (aggregate data collected through Form B)

### Extended data

Harvard Dataverse: Questionnaire for Virtual Mapping in Kenya. https://doi.org/10.7910/DVN/HQRGYT ^22^

This project contains the following extended data:

- FORM MSM Interviews.pdf (Form C)
- FORM Virtual Site Visit Profiling.pdf (Form B)

Data are available under the terms of the Creative Commons Zero "No rights reserved" data waiver (CC0 1.0 Public domain dedication).
