# Supplementary material for: Mapping virtual platforms to estimate the population size of men who have sex with men (MSM) who use internet to find sexual partners: implications to enhance HIV prevention among MSM in Kenya
Source: Gates Open Res. 2020 Dec 10;4:131. Originally published 2020 Sep 16. [Version 2] doi: 10.12688/gatesopenres.13158.2 (PMC7550793; doi:10.12688/gatesopenres.13158.2)
Supplement: Peer review history

# Peer review: Reviewer response for version 1

**DOI:** 10.21956/gatesopenres.14354.r29589

**Published:** 2020-10-12

**Authors:** Keith M. Sabin

**Affiliations:** 1 Joint United Nations Programme on HIV and AIDS (UNAIDS), Geneva, Switzerland

This manuscript describes efforts to systematically develop size estimates of men who have sex with men among those who use social media platforms. The measurement of men who use dating apps or other social media to find is a relatively new and growing area of inquiry. This paper adds a reasonable approach to the toolbox.  

 Specific comments:

- The selection of counties is a bit convenient. To what degree are these counties unusual? Did other counties have polling booth surveys and see smaller measured prevalence? Who participates in polling booth surveys? Are they not people already enrolled in programs and perhaps more likely to know their HIV+ status and therefore more in need of services? Just trying to understand how generalizable these sites are.
- How was the catchment area for WhatsApp or Facebook measured? Is there any geospatial info from those participants?
- I think the formula to account for duplicate accounts seems reasonable. 
- Given that this sampling approach is similar to multi-stage cluster sampling, was there consideration of a design effect in the sample size calculation? 
- I also question if it's reasonable to have one sample size across 3 sites and then show the data for the individual sites. I think the variance should be larger for the individual sites given that the sample size was calculated to be larger for a 95% interval.
- In the final paragraph of the results, there is allusion to "respondents connecting." Is this only digital connection? The authors might want to clarify this though I think the next sentences discussing number of sex partners suggests that these are two different categories of "connection."  
- Is the average number of partners to whom sex was sold based on a denominator of only people who sold sex? Is this number diminished by the 40% who do not sell sex? I think this might be more informative if the numbers were disaggregated by those who did sell sex.
- What proportion of the adult male population do these online groups constitute in each county? It would be interesting to see if the proportions are consistent across counties and have some generalizability.
- This is a strong report and a good contribution to the literature.

Is the work clearly and accurately presented and does it cite the current literature?

Yes

If applicable, is the statistical analysis and its interpretation appropriate?

Yes

Are all the source data underlying the results available to ensure full reproducibility?

Yes

Is the study design appropriate and is the work technically sound?

Yes

Are the conclusions drawn adequately supported by the results?

Yes

Are sufficient details of methods and analysis provided to allow replication by others?

Yes

Reviewer Expertise:

I work on monitoring and evaluating national HIV programs' interventions for key population. I model estimates of the size of HIV epidemics. I study methods for conducting surveys of key populations and estimating their overall sizes.

I confirm that I have read this submission and believe that I have an appropriate level of expertise to confirm that it is of an acceptable scientific standard.

# Response

**Published:** 2020-10-18

**Authors:** Parinita Bhattacharjee

**Affiliations:** University of Manitoba, Kenya

*Response to Reviewer: Dr. Keith Sabin:*

This manuscript describes efforts to systematically develop size estimates of men who have sex with men among those who use social media platforms. The measurement of men who use dating apps or other social media to find is a relatively new and growing area of inquiry. This paper adds a reasonable approach to the toolbox.  

*We thank the reviewer for this acknowledgement.*

Specific comments:

1. The selection of counties is a bit convenient. To what degree are these counties unusual? Did other counties have polling booth surveys and see smaller measured prevalence? Who participates in polling booth surveys? Are they not people already enrolled in programs and perhaps more likely to know their HIV+ status and therefore more in need of services? Just trying to understand how generalizable these sites are.

*As we mentioned, this study was done as part of the routine HIV prevention programming with MSM in Kenya. The three counties represent three distinct geographies in Kenya. Kiambu is located in the east, Kisumu is located in the western part and Mombasa is in the coastal part of Kenya. Polling Booth Surveys have been conducted in 6 counties and the HIV prevalence range from 9% to 27%. These three counties were selected as they represent geographic diversity and HIV prevalence.*

*Polling Booth Surveys use a population based random sampling approach. At the first level hotspots are randomly selected and in the second level MSM who visit the hotspots are randomly selected and screened for eligibility after consent and subsequent interview. So all MSM who visit the sampled hotspots have the possibility of being selected to participate in the survey.*

*In terms of generalizability of the study, the method is generalizable. The findings are presented for each of the county separately and then combined. The same cannot be considered for other counties unless a standard population level parameter is generated using the data.  One such parameter to consider for estimating MSM in similar geographies is the per capita MSM (number of MSM per 1000 adult males), which ranges between 4.1 and 7.2 in the counties studied with an average of 5.9.*

*However, the generalizability of the findings does not affect the main objective of the study, which is to present this approach to locate and estimate the population size of MSM who seek male sexual partners through the virtual sites.*

- How was the catchment area for WhatsApp or Facebook measured? Is there any geospatial info from those participants?

*Thanks for this important comment. WhatsApp and Facebook groups were found to be local to the counties in which this study was conducted. WhatsApp and Facebook groups have no geo-spatial info for the participants, which is a feature only of GSN apps.*

- I think the formula to account for duplicate accounts seems reasonable. 

*Thanks for the comment.*

 

- Given that this sampling approach is similar to multi-stage cluster sampling, was there consideration of a design effect in the sample size calculation? 

*We did not use a design effect for sample size calculation.*

 

- I also question if it's reasonable to have one sample size across 3 sites and then show the data for the individual sites. I think the variance should be larger for the individual sites given that the sample size was calculated to be larger for a 95% interval.

*We wanted to calculate size estimates for each county separately, and that’s why we are showing the results for individual sites.  However, we agree with the comment that the variance would be larger for the individual site, which has contributed to a larger range of estimates. In the revised manuscript we will include the limitation that due to a smaller sample size, the estimate generated can be slightly wider in range.  However, since the mean virtual profiles used in the past month do not vary much between sites, both approach would have given a similar estimate.  Future, a large scale work may include counties with different profiles and therefore can consider a larger sample size in each sites to generate a much precise estimate with narrow range.*

 

- In the final paragraph of the results, there is allusion to "respondents connecting." Is this only digital connection? The authors might want to clarify this though I think the next sentences discussing number of sex partners suggests that these are two different categories of "connection."  

*The connections mentioned in the results are only “digital connections” i.e, other MSM connected through virtual sites and apps. This does not include MSM connected through physical sites as no such information was collected. We will clarify it in the revised version of the manuscript.*


* *

- Is the average number of partners to whom sex was sold based on a denominator of only people who sold sex? Is this number diminished by the 40% who do not sell sex? I think this might be more informative if the numbers were disaggregated by those who did sell sex.

*Thanks for the comment.  The data presented on the average number of partners to whom sex was sold (money involved) is included in the total denominator.  Though we did not present this by those who sell sex and who do not, we reviewed and is informative as you suggested. We will add it in the revised manuscript. (The format did not allow us to add a table in the response).*

- What proportion of the adult male population do these online groups constitute in each county? It would be interesting to see if the proportions are consistent across counties and have some generalizability.

*Thanks for this excellent comment. We calculated the standard measure of per capita, number of online MSM per 1000 adult (15-64 years) males.  It shows that the per capita MSM online per 1000 adult males are 6.7, 7.2 and 4.1 for Kiambu, Kisumu and Mombasa respectively. We will add this in the revised version of the manuscript.*

- This is a strong report and a good contribution to the literature.

*Thanks for the acknowledgement*

# Peer review: Reviewer response for version 1

**DOI:** 10.21956/gatesopenres.14354.r29592

**Published:** 2020-10-08

**Authors:** Rajatashuvra Adhikary

**Affiliations:** 1 National Professional Officer (HIV/AIDS), HIV Surveillance, WHO India Country Office, New Delhi, Delhi, India

It is an excellent piece of work by an outstanding group of researchers. This work will be immensely helpful in mapping and estimating the size of key populations at the virtual space. The best part of this paper is that it is simple in its approach and language. However, i do have the following key observations: 

1. The conclusion section highlights, "..there is a clear indication that use of geo-spot based mapping alone underestimates the total number of MSM in a given context.." Similarly, an earlier part of the paper rightly mentions, "Since that methodology (geo-spot based mapping or programmatic mapping and size estimation exercise) did not include MSM who use virtual platforms, there could have been an underestimation of total number of MSM". 
  But, if we compare the current MSM estimates with the previous estimates (given in the paper), the size of MSM in Mombasa declined by 48%, in Kismu by 37% and across three counties by 5%. Whereas, it was expected to increase at least by 26% (as 26% of the respondents exclusively using virtual platforms). In fact, 118% increase in size of MSM only in one of the three counties (Kiambu) need further explanations or justifications.
2. Beside the 'selection bias', perhaps, there is another issue with the selection of 435 eligible respondents for the survey. Although the required number of virtual sites (15) within a county and the required number of respondents (10) within each selected virtual site was selected randomly, the adopted design did not produce a self-weighted sampling design due to different number of total virtual sites within a county and different number of total eligible respondents within each selected virtual site. Not carrying out an weighted analysis could be highlighted as one of the limitations. 
3. The data collection was carried out by CBO members and program staff. It is important to mention how 'objectivity' as well as 'quality' of data was strictly maintained through rigorous monitoring and supervision mechanism. It is important to note.                   

Is the work clearly and accurately presented and does it cite the current literature?

Yes

If applicable, is the statistical analysis and its interpretation appropriate?

Yes

Are all the source data underlying the results available to ensure full reproducibility?

Yes

Is the study design appropriate and is the work technically sound?

Yes

Are the conclusions drawn adequately supported by the results?

Partly

Are sufficient details of methods and analysis provided to allow replication by others?

Yes

Reviewer Expertise:

I am working in the areas of research, surveillance and monitoring & evaluation for last 25+ years. I was involved with several rounds of BSS, IBBS, HSS, p-MPSE etc.

I confirm that I have read this submission and believe that I have an appropriate level of expertise to confirm that it is of an acceptable scientific standard.

# Response

**Published:** 2020-10-17

**Authors:** Parinita Bhattacharjee

**Affiliations:** University of Manitoba, Kenya

*Response to Reviewer Comments (Dr. R Adhikary):*

It is an excellent piece of work by an outstanding group of researchers. This work will be immensely helpful in mapping and estimating the size of key populations at the virtual space. The best part of this paper is that it is simple in its approach and language.

*Thank you very much for your response and kind words.*

However, I do have the following key observations: 

1. The conclusion section highlights, “there is a clear indication that use of geo-spot based mapping alone underestimates the total number of MSM in a given context." Similarly, an earlier part of the paper rightly mentions, "Since that methodology (geo-spot based mapping or programmatic mapping and size estimation exercise) did not include MSM who use virtual platforms, there could have been an underestimation of total number of MSM". But, if we compare the current MSM estimates with the previous estimates (given in the paper), the size of MSM in Mombasa declined by 48%, in Kismu by 37% and across three counties by 5%. Whereas, it was expected to increase at least by 26% (as 26% of the respondents exclusively using virtual platforms). In fact, 118% increase in size of MSM only in one of the three counties (Kiambu) need further explanations or justifications.

*Thank you for your comment.  It was referenced in the paper that the geo-mapping or programmatic mapping under estimate the size of MSM population as they do not account for those exclusively using virtual sites. The findings presented in this paper is only from mapping and size estimation in the virtual sites. The findings show that overall (3 counties together) 74% of the virtual users visited a physical location.  This suggests that 26% of MSM who seek partners in virtual sites do not visit a physical location and hence are missed when mapping and estimation takes place using a geo-mapping/programmatic mapping approach. The size estimates presented in the results are derived from mapping and size estimation in virtual sites and hence cannot be considered as final size estimates of “all MSM” in the targeted counties. The paper is trying to communicate that it is critical to also map MSM in the virtual sites as geographic mapping may underestimate the population size considering the finding that 26% of MSM do not visit hotspots which geographic mapping cover. Hence we cannot say that numbers of MSM in the 3 counties actually declined. It needs to be considered that we have size estimates from the counties using geo-mapping method and size estimates from the same counties using virtual mapping method and both map and estimate different sites. The findings suggest that some MSM visit only physical hotpots/ sites, some visit only virtual sites and some visit both. While there is an overlap, there is also a sub population which visit only one type of site. Hence, we suggest that the total estimated size of MSM population in a county would be the number estimated using a programmatic mapping approach and the number estimated using a virtual mapping approach after subtracting the overlap, if both the mapping is done around the same time period.  The main objective of this paper is not to show the overall size estimate of MSM in the counties, but to present the methodology of virtual mapping, share the findings which is the size estimate of MSM in virtual sites and the overlap with MSM visiting physical sites/ hotspots.*

**

2. Beside the 'selection bias', perhaps, there is another issue with the selection of 435 eligible respondents for the survey. Although the required number of virtual sites (15) within a county and the required number of respondents (10) within each selected virtual site was selected randomly, the adopted design did not produce a self-weighted sampling design due to different number of total virtual sites within a county and different number of total eligible respondents within each selected virtual site. Not carrying out a weighted analysis could be highlighted as one of the limitations. 

*Thanks for the comment on an unweighted analysis.  We recognize this. We have estimated the size of virtual MSM at the county level after accounting for the overlap within the county and aggregated county level estimates to produce the overall estimate for the 3 counties. Therefore, the estimate has not been affected by a county weight, though the equal sample size in virtual sites might have affected if the profile of MSM differed across virtual sites.  However, since a very large proportion of virtual MSM have profiles in multiple virtual sites, an unweighted analysis might not have affected the estimates.  The profile of MSM presented at the overall level for 3 counties together might have affected due to an unweighted analysis. We have noted this point and will clarify it in the revised version of the paper.*

**

3. The data collection was carried out by CBO members and program staff. It is important to mention how 'objectivity' as well as 'quality' of data was strictly maintained through rigorous monitoring and supervision mechanism. It is important to note. 

*Thanks for pointing this out. The data collectors were trained by UoM and were monitored regularly by UoM and its Kenyan partner, Partners for Health and Development in Africa (PHDA). Onsite monitoring and supervision was conducted every week during the data collection process. Data quality checks were done on a regular basis by UoM and PHDA staff. We will add this in the revised version of the manuscript.*
